# A delayed presentation of a giant traumatic pseudocyst of the pancreas

**DOI:** 10.1093/jscr/rjag239

**Published:** 2026-04-09

**Authors:** Yasmina Yassine, Siham Sbihi, Khaoula Haij, Hala Aouroud, Oussama Nacir, Fatima Ezzahra Lairani, Adil Ait Errami, Sofia Oubaha, Zouhour Samlani, Khadija Krati

**Affiliations:** Department of Gastroenterology, University Hospital Center Mohammed VI, Faculty of Medicine and Pharmacy, Cadi Ayyad University, Marrakech 40000, Morocco; Department of Gastroenterology, University Hospital Center Mohammed VI, Faculty of Medicine and Pharmacy, Cadi Ayyad University, Marrakech 40000, Morocco; Department of Gastroenterology, University Hospital Center Mohammed VI, Faculty of Medicine and Pharmacy, Cadi Ayyad University, Marrakech 40000, Morocco; Department of Gastroenterology, University Hospital Center Mohammed VI, Faculty of Medicine and Pharmacy, Cadi Ayyad University, Marrakech 40000, Morocco; Department of Gastroenterology, University Hospital Center Mohammed VI, Faculty of Medicine and Pharmacy, Cadi Ayyad University, Marrakech 40000, Morocco; Department of Gastroenterology, University Hospital Center Mohammed VI, Faculty of Medicine and Pharmacy, Cadi Ayyad University, Marrakech 40000, Morocco; Department of Gastroenterology, University Hospital Center Mohammed VI, Faculty of Medicine and Pharmacy, Cadi Ayyad University, Marrakech 40000, Morocco; Department of Gastroenterology, University Hospital Center Mohammed VI, Faculty of Medicine and Pharmacy, Cadi Ayyad University, Marrakech 40000, Morocco; Department of Gastroenterology, University Hospital Center Mohammed VI, Faculty of Medicine and Pharmacy, Cadi Ayyad University, Marrakech 40000, Morocco; Department of Gastroenterology, University Hospital Center Mohammed VI, Faculty of Medicine and Pharmacy, Cadi Ayyad University, Marrakech 40000, Morocco

**Keywords:** abdominal trauma, pancreatic trauma, pancreatic pseudocyst

## Abstract

Pancreatic trauma is an uncommon but potentially severe injury that can lead to serious complications such as pseudocyst formation. Optimal management remains unclear due to limited evidence. A 19-year-old male presented with progressive abdominal pain 15 days after blunt abdominal trauma. Imaging revealed a giant pancreatic pseudocyst (30 × 21.5 cm) secondary to a pancreatic laceration. Surgical cystogastrostomy was performed, draining 4700 mL of fluid. The patient had an uneventful recovery and remained symptom-free at 1-year follow-up. This case underscores the challenges in managing pancreatic pseudocysts following trauma. While endoscopic and percutaneous techniques are effective in selected cases, open surgical approaches remain appropriate in cases with complex anatomy. Further research is needed to guide treatment decisions.

## Introduction

Traumatic injury of the pancreas is rare, accounting for approximately 3%–12% of intra-abdominal trauma cases. However, it is associated with significant morbidity and mortality, including complications such as hemorrhage, pseudocyst and fistula formation, and pancreatitis [1, 2]. Pseudocysts are among the most common and serious complications. They refer to an encapsulated collection of homogeneous fluid with little or no necrotic tissue located near the pancreas.

Management options include surgical, percutaneous, and endoscopic approaches. However, the absence of a clear consensus on the optimal treatment strategy remains a major challenge [3]. This uncertainty is largely due to the scarcity of large-scale studies and the limited clinical experience reported in the literature.

Furthermore, delays in intervention for patients who require it have been shown to increase both morbidity and mortality [4].

Here, we present a rare case of a young patient with isolated blunt pancreatic trauma complicated by a giant pseudocyst.

## Case presentation

A 19-year-old male patient, with no relevant medical history, presented to the emergency department 15 days after an assault where he suffered multiple kicks in his abdomen. He had been experiencing worsening epigastric pain since the initial aggression. He also complained of nausea and several episodes of nonbilious, nonhemorrhagic vomiting.

**Figure 1:**
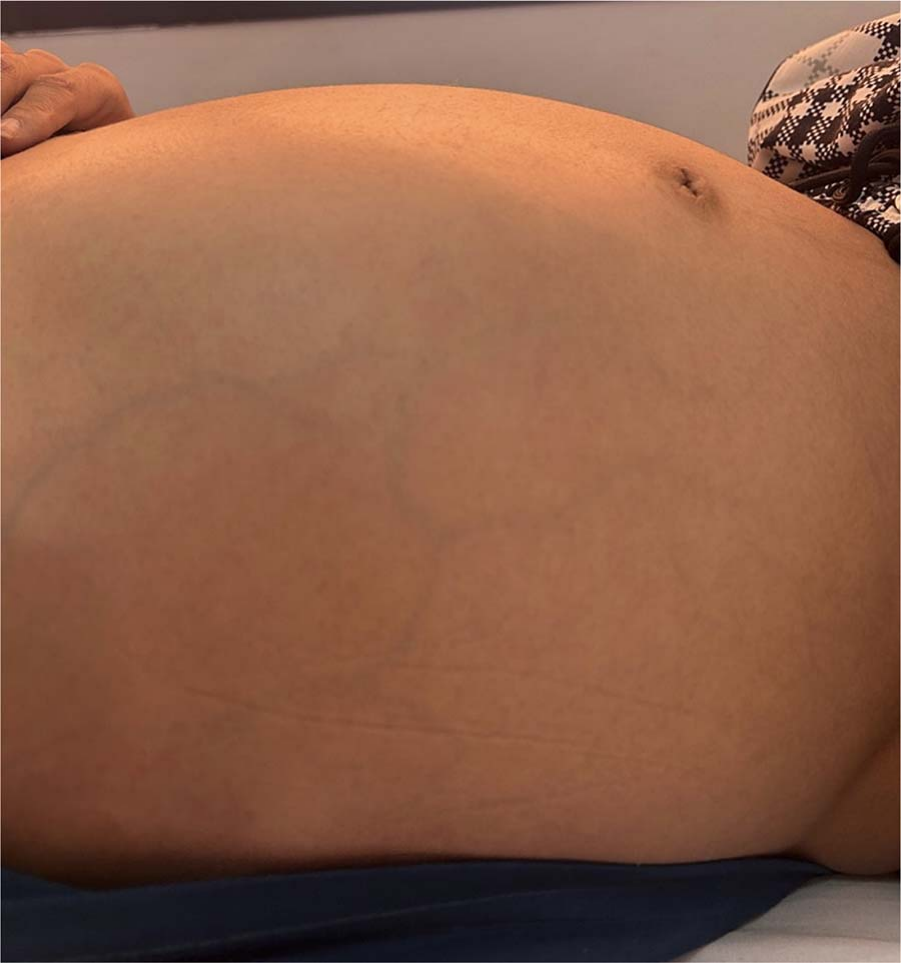
Important abdominal distension.

Upon examination, his vitals were stable, with an important abdominal distension (Fig. 1). Palpation found an exquisite tenderness in the epigastrium and the left upper quadrant. There was no evidence of flank or periumbilical bruising.

Blood tests showed high serum lipase (588 U/L) and serum amylase (1603 U/L). His liver function was normal, with an elevated C-reactive protein to 116 mg/L.

**Figure 2:**
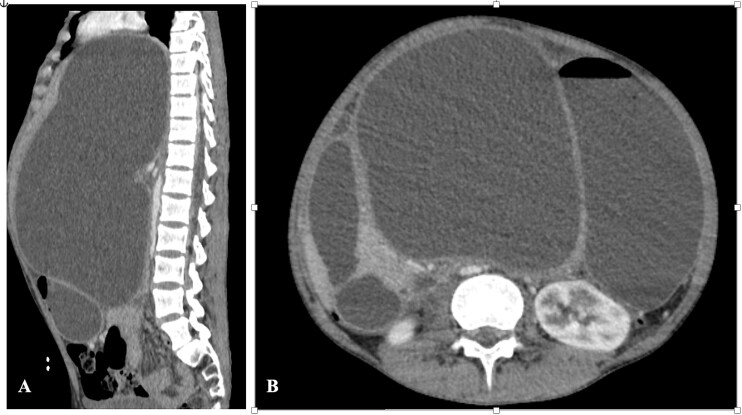
CT abdomen—(A) sagittal section and (B) axial section showing the pseudocyst with significant organ compression.

A computed tomography (CT) scan of the abdomen demonstrated a deep laceration of the body and the neck of the pancreas, with a large intrapancreatic collection displacing the liver, stomach, and inferior vena cava, with regular and well-defined contours, measuring 30 × 21.5 cm (Fig. 2).

There was no evidence of injury to any of the surrounding structures including vascular axes.

**Figure 3:**
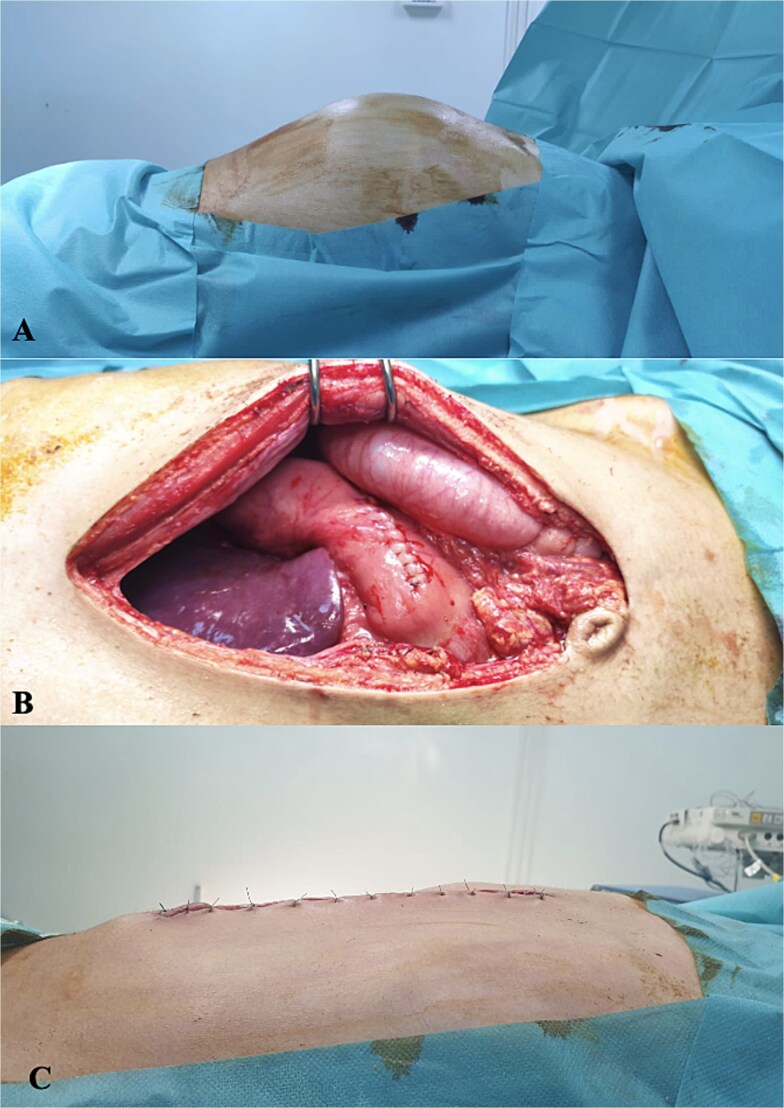
(A) Preoperative distension of the abdomen, (B) anastomosis of posterior wall of stomach and cyst wall, and (C) postoperative outcome.

Therefore, an exploratory laparotomy was performed. The intraoperative finding was a huge cystic swelling occupying the upper abdomen with 4700 mL of serous fluid which was drained and cystogastrostomy was performed (Fig. 3). His postoperative care was uneventful and he was discharged after a week. He remained well and symptom-free a year postoperatively during follow-up.

## Discussion

Pancreatic injuries are classified according to the extent of parenchymal and ductal damage, with the American Association for the Surgery of Trauma (AAST) pancreatic Organ Injury Scale (OIS), which is commonly used to guide management in hemodynamically stable patients based on CT findings [5]. Early identification of main pancreatic duct disruption is crucial, as it is a primary contributor to delayed complications such as pseudocyst formation [6].

Pancreatic pseudocysts may arise following acute or chronic pancreatitis or, less commonly, as a complication of pancreatic trauma, as demonstrated in the present case. Although their reported incidence is low, estimated at 0.5–1 per 100 000 adults per year, pseudocysts remain a clinically significant source of morbidity [7].

Historically, pancreatic pseudocysts were managed with open surgical internal drainage. With advances in minimally invasive techniques, percutaneous, endoscopic, and laparoscopic approaches are increasingly utilized. The choice of intervention, however, depends largely on cyst characteristics, anatomical relationships, and available expertise.

In our case, open surgical cystogastrostomy was selected due to the challenging anatomical configuration of the pseudocyst, which was closely adherent to the posterior wall of the stomach, allowing for the creation of a wide and dependent internal drainage under direct visualization. Although endoscopic ultrasound (EUS)-guided drainage is an effective minimally invasive option for pseudocysts abutting the stomach or duodenum, this modality was not available at our institution at the time of management. Percutaneous drainage was also considered less favorable given the risk of prolonged external catheterization and the potential for external pancreatic fistula formation.

EUS-guided drainage has been shown to be highly effective for appropriately selected pseudocysts, particularly those adjacent to the stomach or duodenum, as highlighted in the systematic review by Teoh et al. [6]. Randomized controlled trials have demonstrated comparable success rates between EUS-guided cystogastrostomy and surgical cystogastrostomy, with shorter hospital stays, reduced costs, and improved quality-of-life scores favoring the endoscopic approach [8]. Nevertheless, open surgical cystogastrostomy remains a valid and effective option in selected cases with unfavorable anatomy or when minimally invasive approaches are deemed suboptimal, avoiding the need for an external catheter with a reduced risk of external pancreatic fistula formation.

Further randomized studies with larger patient cohorts and long-term follow-up are required to better define the optimal management strategy and establish standardized clinical guidelines.

## Conclusion

Traumatic pseudocysts of the pancreas are rare but serious complications requiring timely detection and appropriate management. While minimally invasive techniques are increasingly preferred, surgical cystogastrostomy remains a reliable option in selected cases.
